# Stent Thrombosis is the Primary Cause of ST-Segment Elevation Myocardial Infarction following Coronary Stent Implantation: A Five Year Follow-Up of the SORT OUT II Study

**DOI:** 10.1371/journal.pone.0113399

**Published:** 2014-11-19

**Authors:** Søren Lund Kristensen, Anders M. Galløe, Leif Thuesen, Henning Kelbæk, Per Thayssen, Ole Havndrup, Peter Riis Hansen, Niels Bligaard, Kari Saunamäki, Anders Junker, Jens Aarøe, Ulrik Abildgaard, Jørgen L. Jeppesen

**Affiliations:** 1 Department of Cardiology, Copenhagen University Hospital Roskilde, Roskilde, Denmark; 2 Department of Cardiology, Copenhagen University Hospital Gentofte, Hellerup, Denmark; 3 Department of Medicine, Aarhus University Hospital Herning, Herning, Denmark; 4 The Heart Centre, Copenhagen University Hospital Rigshospitalet, Copenhagen, Denmark; 5 Department of Cardiology, Odense Universy Hospital, Odense, Denmark; 6 Department of Cardiology, Copenhagen University Hospital Bispebjerg, Copenhagen, Denmark; 7 Department of Cardiology, Aalborg University Hospital, Aalborg, Denmark; 8 Department of Medicine, Copenhagen University Hospital Glostrup, Glostrup, Denmark; University Medical Center Utrecht, Netherlands

## Abstract

**Background:**

The widespread use of coronary stents has exposed a growing population to the risk of stent thrombosis, but the importance in terms of risk of ST-segment elevation myocardial infarctions (STEMIs) remains unclear.

**Methods:**

We studied five years follow-up data for 2,098 all-comer patients treated with coronary stents in the randomized SORT OUT II trial (mean age 63.6 yrs. 74.8% men). Patients who following stent implantation were readmitted with STEMI were included and each patient was categorized ranging from definite- to ruled-out stent thrombosis according to the Academic Research Consortium definitions. Multivariate logistic regression was performed on selected covariates to assess odds ratios (ORs) for definite stent thrombosis.

**Results:**

85 patients (4.1%), mean age 62.7 years, 77.1% men, were admitted with a total of 96 STEMIs, of whom 60 (62.5%) had definite stent thrombosis. Notably, definite stent thrombosis was more frequent in female than male STEMI patients (81.8% vs. 56.8%, p = 0.09), and in very late STEMIs (p = 0.06). Female sex (OR 3.53 [1.01–12.59]) and clopidogrel (OR 4.43 [1.03–19.01]) was associated with increased for definite stent thrombosis, whereas age, time since stent implantation, use of statins, initial PCI urgency (STEMI [primary PCI], NSTEMI/unstable angina [subacute PCI] or stable angina [elective PCI]), and glucose-lowering agents did not seem to influence risk of stent thrombosis.

**Conclusion:**

In a contemporary cohort of coronary stented patients, stent thrombosis was evident in more than 60% of subsequent STEMIs.

## Introduction

Stent thrombosis is a rare but serious complication following coronary stenting, associated with a high risk of acute coronary artery closure, ST-segment elevation myocardial infarction (STEMI) and sudden cardiac death [1], [2]. Up until recently, a growing number of patients has been treated with coronary stents, leaving them exposed to the risk of stent thrombosis [3]. This was reflected in a recent study of consecutive STEMI patients, where the number of STEMIs resulting from stent thrombosis nearly doubled (6% to 11%) in the period from 2003–10 [4].

Despite these findings, information on the percentage of STEMIs caused by stent thrombosis is sparse, and most studies on the topic are hampered by short follow-up [5]. The primary goal of the present study was to evaluate the prevalence of stent thrombosis in patients presenting with STEMI after percutaneous coronary intervention (PCI) during long term follow-up, and additionally, to identify clinical predictors of stent thrombosis in these subjects. For these analyses, we examined data from 2098 patients treated with coronary stents in the SORT OUT II trial [5].

## Methods

### Study population

The Danish Organization on Randomized Trials with clinical Outcome (SORT OUT) is an independent clinical cardiovascular research collaboration among the five Danish centers performing coronary stent implantations. In the present study we used follow-up data from the SORT OUT II trial, which in the period 2004–2006, randomized 2098 patients eligible for percutaneous coronary intervention (PCI), to one of the first two commercially available drug-eluting stents; the sirolimus-eluting Cypher stent (Cordis/Johnson & Johnson, Florida) or the paclitaxel-eluting Taxus stent (Boston Scientific Group, Massachusetts).^5^ Each citizen in Denmark is provided with a unique and permanent civil registration number, and by use of this number all in- and outpatient hospital admissions, deaths and emigrations are reported to national registries and identifiable from these sources.

We assessed all SORT OUT II participants irrespective of randomization as there were no short- or long-term differences in the risk of major adverse cardiovascular events and stent thrombosis when comparing the two stents [5], [6]. The SORT OUT II cohort was followed from stent implantation until death, migration, or five years from study inclusion. Patients hospitalized with one (or more) STEMI(s) during follow-up comprised the present study cohort.

### Outcome – stent thrombosis probability

All STEMI admissions following SORT OUT II study inclusion were identified by case notes, electrocardiogram (ECG) findings, and cardiac biomarkers. Each STEMI was then categorized as definite-, probable-, possible- or ruled out stent thrombosis, according to the classification defined by the Academic Research Consortium (ARC) [7].

Categorization of stent thrombosis probability was based on detailed records of hospital admission, including case notes, ECG findings, and angiographic findings. Information on out-of hospital deaths were obtained from general practitioners’ records and out-patients coronary artery angiographies were evaluated from the records and/or by inspection of copies of the angiographic recordings. Classification as definite stent thrombosis demanded either angiographic or autopsy confirmation. In order to resemble a real-life scenario of stent thrombosis risk in coronary stented patients admitted with STEMI, we also included information on non-randomized stents unrelated to the SORT OUT II study in our stent thrombosis evaluation. Finally, we merged probable- and possible stent thrombosis into one group (possible stent thrombosis) and further we stratified the STEMIs in three groups according to time passed since stent implantation; early (0–30 days), late (31–365 days) and very late stent thrombosis (>365 days). All the above outcomes, including ECGs and angiograms, and also the specific causes of cardiac and non- cardiac deaths, were adjudicated by the independent SORT OUT II adjudication committee, as described in details elsewhere [5]. The basis of the cause of death adjudication was the main underlying disease causing death. In most cases, the cause of death was cancer (n = 67), and in these cases, patients were adjudicated not to have stent thrombosis.

### Medical treatment

From patient records, we assessed each patients use of the following medications at time of STEMI (ATC codes); aspirin (B01AC06, N02BA01), clopidogrel (B01AC04), vitamin K antagonists (B01AA), angiotensin-converting enzyme inhibitors (ACE-Is)/angiotensin receptor blockers (ARBs) (C09), beta-blockers (C07), loop diuretics (C03C), proton-pump inhibitors (A02BC), calcium channel antagonists (C08), nitrates (C01DA) and glucose-lowering agents (A10). All patients in SORT OUT II were prescribed 12 months of dual antiplatelet treatment (low dose aspirin and clopidogrel) and monotherapy with aspirin onwards after PCI. We did not have information on the overall compliance in the SORT OUT II cohort, but a prior registry-based Danish study on post-MI treatment adherence showed that approximately 80% were treated with dual antiplatelet therapy for one year following PCI [8]. Among the 31 SORT OUT II patients who were admitted with a STEMI within 12 months of initial stent implantation, none were treated with vitamin K antagonists, 18 (58.1%) received the recommended dual antiplatelet therapy, 10 (32.3%) were treated with aspirin monotherapy and the remaining 3 (9.6%) patients received no antithrombotic therapy.

### Statistics

Baseline characteristics for the study population were summarized as means with standard deviations for continuous variables and frequencies and percentages for categorical variables. For descriptive statistics we used Chi-square tests for categorical data and Kruskall-Wallis test for non-parametric data. We fitted a multivariate logistic regression model to estimate odds ratios (ORs) for the probability of definite stent thrombosis, as compared to possible or ruled out stent thrombosis among patients subsequently admitted with STEMI. The model included adjustments for; sex, age (divided into inter-quartile ranges [IQR]), time since PCI (categorized as early [<30 days], late [31–364 days] or very late [>365 days]), initial PCI urgency (elective vs. subacute or primary [acute] PCI), and pharmacological treatment with aspirin, clopidogrel, and antidiabetics. As a sensitivity analysis, we included cases of unexplained death (n = 52), which according to ARC criteria corresponds to probable stent thrombosis if death occurs within 30 days of PCI, and possible stent thrombosis if more than 30 days after PCI. In the sensitivity analysis, we estimated odds ratios for definite *or* probable stent thrombosis as opposed to definite stent thrombosis alone in the primary analysis.

### Ethics

The SORT OUT II study was conducted in accordance with the second Helsinki declaration and the local biomedical research committee (Health Research Ethical Committee of the Capital Region, Denmark) approved the study. Trial registration: clinicaltrials.gov, identifier: NCT00388934. All study participants provided written informed consent.

## Results

**Figure 1:**
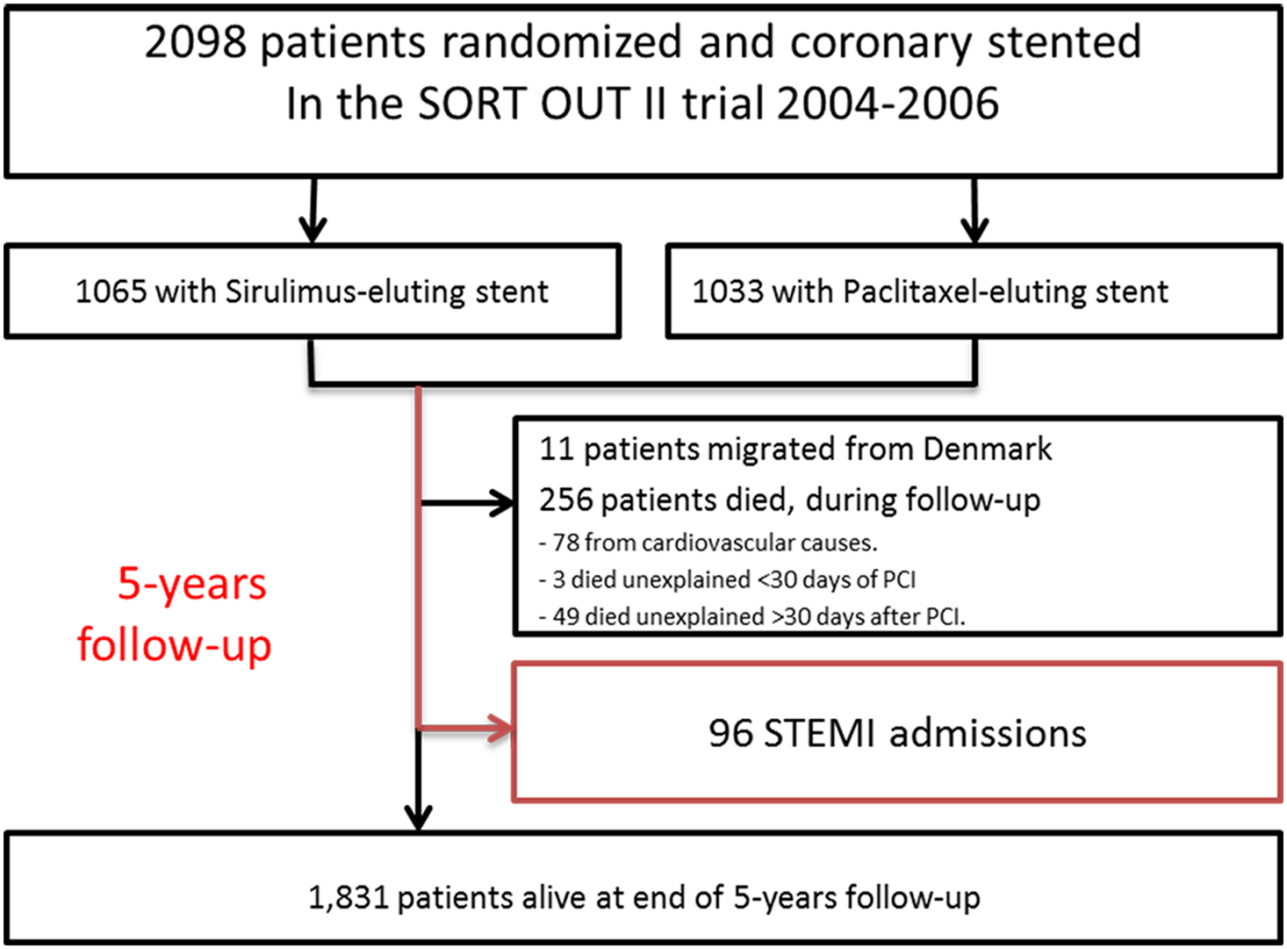
Flowchart of the STEMI-subgroup study population. STEMI – ST segment elevation myocardial infarction.

**Figure 2:**
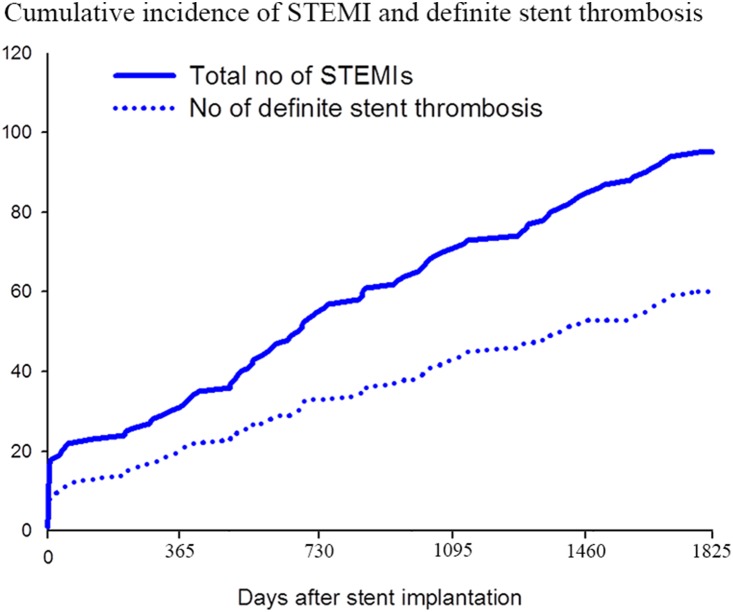
Fraction of STEMI’s caused by definite stent thrombosis according to time from stent implantation. STEMI - ST segment elevation myocardial infarction.

**Figure 3:**
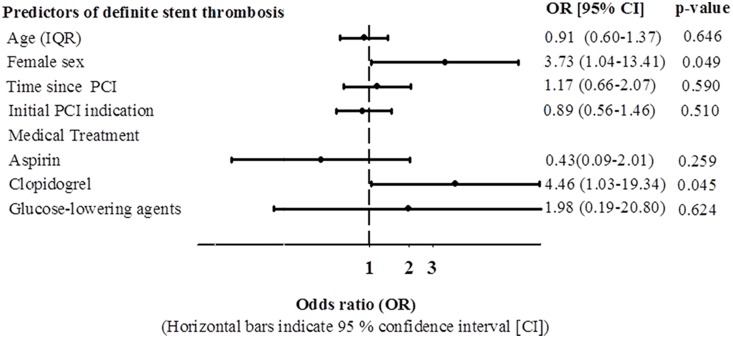
Odds ratios for definite stent thrombosis according to presence of selected covariates. STEMI - ST segment elevation myocardial infarction, OR – Odds ratio, CI – confidence interval.

Within 5 years of coronary stent implantation, we identified 85 patients (4.1%) who were admitted with a total of 96 STEMIs (Figure 1). Definite stent thrombosis was confirmed in 60 STEMIs (62.5%) whereas 14 STEMIs (13.5%) were classified as probable or possible stent thrombosis and in the remaining 22 STEMIs (24.0%) stent thrombosis was ruled out i.e. lesions were not related to a prior stented area (de novo lesions). In 13 patients the categorization as definite- or possible stent thrombosis was related to a non-randomized stent, i.e. stent(s) that were implanted before or after the patient was randomized in the SORT OUT II trial. General characteristics of the STEMI population stratified by stent thrombosis probability are summarized in Table 1. The mean age was 62.7 (SD 11.0) years and 77.1% were men. Notably, STEMI resulting from definite stent thrombosis appeared to be more common in women than in men (81.8% vs. 56.8%, p = 0.09), and more than a year after stent implantation (p = 0.06), whereas we found no relation between initial PCI indication at randomization and stent thrombosis probability (p = 0.56). A total of 11 patients suffered a second STEMI during follow-up, of whom five had definite stent thrombosis, five had recurring definite stent thrombosis, and one had a de novo lesion. The cumulative number of STEMI and definite stent thrombosis cases according to time passed since study inclusion is shown in Figure 2. We compared the use of cardiovascular medication at time of STEMI stratified by the presence of stent thrombosis, and observed no significant differences, except for the use of dual anti-platelet therapy which was higher in the definite stent thrombosis group (p = 0.03). Use of glucose-lowering agents also seemed more frequent in patients with definite stent thrombosis but the difference was non-significant (p = 0.15). In a multivariate logistic regression model (Figure 3) female sex was associated with a significantly increased risk of definite stent thrombosis (OR 3.73 [95% CI 1.04–13.41]), as well as use of clopidogrel (OR 4.46 [1.03–19.34]). There was a non-significant indication towards a protective effect of aspirin use (OR 0.43 [0.09–2.01]), and no association to age, initial PCI urgency, time since PCI and glucose-lowering agents. Of note, from a total of 256 deaths which occurred during 5 year follow-up, 52 patients (3 within 30 days of PCI) died of unexplained causes (Figure 1). We did not include these cases of unexplained death in our primary analysis, although we included them in a sensitivity analysis. In this sensitivity analysis where we examined the risk of definite *or* probable stent thrombosis, odds ratios were consistent with findings of the primary analysis, although ongoing use clopidogrel was no longer associated with a significantly increased risk of (definite and probable) stent thrombosis (not shown).

**Table 1:** General characteristics of patients at time of admission for STEMI.

	Total	Definite stentthrombosis	Probable or possiblestent thrombosis	No stentthrombosis	P-value
**No. of STEMIs (%)**	96 (100)	60 (62.5)	13 (13.5)	23 (24.0)	
**Age, mean (SD)/years**	62.7 (11.0)	62.3 (12.0)	61.6 (11.0)	64.1 (8.3)	0.42
**Male, (%)**	74 (77.1)	42 (56.8)	11 (14.9)	21 (28.4)	0.09
**Female (%)**	22 (22.9)	18 (81.8)	2 (9.1)	2 (9.1)	
**Initial PCI urgency (%)**					0.56
**Primary PCI**	24 (25.0)	14 (23.3)	2 (15.4)	8 (34.8)	
**Subacute** *	36 (37.5)	22 (36.6)	7 (53.8)	7 (30.4)	
**Elective**	36 (37.5)	24 (40.0)	4 (30.8)	8 (34.8)	
**Days to STEMI/ST (%)**					0.06
**Early (0–30)**	18 (18.8)	9 (15.0)	1 (7.7)	8 (34.8)	
**Late (31–365)**	13 (13.5)	11 (18.4)	0 (0)	2 (8.7)	
**Very late (>365)**	65 (67.7)	40 (66.6)	12 (92.3)	13 (56.5)	
**Medical treatment (%)**					
**Aspirin**	86 (89.6)	53 (88.3)	12 (92.3)	21 (91.3)	0.87
**Clopidogrel**	20 (20.8)	17 (28.3)	0 (0)	3 (13.0)	0.03
**Dual antiplatelet therapy**	20 (20.8)	17 (28.3)	0 (0)	3 (13.0)	0.03
**Vitamin K antagonists**	6 (6.3)	4 (6.7)	0 (0)	2 (8.7)	0.71
**Statins**	76 (79.2)	46 (76.7)	11 (84.6)	19 (82.6)	0.73
**ACE-I/ARBs**	44 (45.8)	30 (50.0)	4 (30.8)	10 (43.5)	0.44
**Beta-blockers**	64 (66.7)	40 (66.7)	8 (61.5)	16 (69.6)	0.89
**Diuretics**	30 (31.3)	19 (31.7)	4 (30.8)	7 (30.4)	0.99
**Protein pump inhibitors**	19 (19.8)	14 (23.3)	2 (15.4)	3 (13.0)	0.52
**Ca-antagonists**	17 (17.7)	9 (15.0)	3 (23.1)	5 (21.7)	0.67
**Nitrates**	15 (15.6)	12 (20.0)	1 (7.7)	2 (8.7)	0.31
**Glucose-lowering agents**	10 (10.4)	9 (15.0)	0 (0)	1 (4.3)	0.15

ST = stent thrombosis, ACE-I = Angiotensin-converting enzyme inhibitors, ARBs = angiotensin receptor blockers. PPI = Proton pump inhibitors. Dual antiplatelet therapy = clopidogrel and aspirin.

*Subacute PCI – patients with NSTEMI or unstable angina pectoris.

## Discussion

In this study of a large population of all-comers treated with coronary stents, the number of patients re-admitted with STEMI within five year of follow-up was low. However, in more than 60% of STEMIs patients had definite stent thrombosis. The majority of stent thrombosis occurred very late (>365 days after initial stent implantation). Thus, our data imply that secondary preventions strategies work reasonably well to prevent STEMI culprits in non-PCI treated segments of the coronary vasculature, but also that implanted stents endure as vulnerable areas of the coronary arteries.

In a multivariate analysis comparing STEMIs with and without evidence of stent thrombosis, we failed to find an association between initial PCI urgency (elective vs. subacute or acute) and stent thrombosis. This together with the somewhat surprising finding of an association between stent thrombosis and DAPT was in contrast to a previous study of stent thrombosis predictors [2], [9], [10]. In fact, the association between DAPT and definite stent thrombosis was primarily observed in patients (n = 65) who suffered a STEMI more than 12 months after stent implantation. Among these patients, 13 received DAPT, of whom 12 presented with STEMIs caused by definite stent thrombosis. It is likely that the latter patients were considered by treating physicians to be at higher risk as DAPT had been continued beyond the recommended 12 months, albeit that as indicated in Table 1, there were no apparent differences in measured potential risk factors for stent thrombosis, e.g. index PCI urgency (acute coronary syndrome vs. stable angina) or diabetes prevalence as measured by treatment with glucose-lowering drugs.

We also observed a trend towards a protective effect of ongoing aspirin treatment, and a significantly elevated OR for definite stent thrombosis in female STEMI subjects. We have no obvious explanation for the higher risk of stent thrombosis in females and the finding warrants confirmation from other studies.

We chose not to include cases of unexplained death in our primary analysis as our intention was to focus on definite stent thrombosis alone, and a more clinical perspective of previously coronary stented patients admitted with STEMI. However, we included cases of unexplained deaths in a sensitivity analysis and found results comparable to those of the primary analysis. Our finding of definite-, probable- or possible stent thrombosis in 7.4% of the study subjects after 5-year follow-up corresponded quite well with a large meta-analysis, although the median follow-up in that analysis was 22 months [10]. Indeed, in the current study we demonstrated that the rate of stent thrombosis continued at a relatively constant rate beyond the first two years after stent implantation.

Studies of first generation drug-eluting stents in clinical practice with unselected patients demonstrated higher rates of stent thrombosis compared to the randomized trials, which emphasized the importance of dual antiplatelet treatment after implantation of drug-eluting stents [1], [10]–[12]. The comprehensive meta-analysis by Palmerini et al. further showed that the majority of studies, similar to SORT OUT II, found non-inferiority when comparing the sirolimus and paclitaxel eluting stents.

Studies comparing prognosis after stent thrombosis induced STEMI vs. de novo induced lesion STEMI have shown higher risk in regards of mortality, risk of recurrent myocardial infarction and post-PCI rates of major adverse cardiovascular events following stent thrombosis [13]–[15]. Along this line, a 7-year outcome with fractional flow reserve (FFR)-guided PCI, showed a significantly reduced risk for recurrent MI when PCI were deferred by FFR vs. performed due to PCI [16]. This reduced risk might be due to potential PCI-related damage or atherosclerotic disease being more advanced in previously stented segments. The LEADERS trial, which compared biolimus- and sirolimus-eluting stents showed a significant reduction in very late stent thrombosis (>1 year) for the biolimus-stent [17]. Contrarily, the SORT OUT V study which was a similar comparison of the biolimus and sirolimus stents, found no improvement in regard of a combined endpoint of cardiac death, myocardial infarction and stent thrombosis [18]. Interestingly, a significantly increased risk of definite stent thrombosis was observed among patients treated with the biolimus-stent, which may indicate that the risk of stent thrombosis and its marked contribution to subsequent STEMI cases observed in the present study remains relevant for patients treated with newer generations of drug-eluting stents.

### Conclusions

In a large unselected PCI population treated with coronary stents, the incidence of subsequent STEMIs was low within five years of follow-up. However, stent thrombosis was present in more than 60% of STEMIs in these patients with one or more previously implanted stents, and the risk of stent thrombosis was higher beyond the first year following stent implantation. Continued focus on reduction of stent thrombosis, particularly beyond the first year after PCI is warranted.
